# Diet, dementia, and the hippocampus

**DOI:** 10.1093/stcltm/szaf007

**Published:** 2025-05-19

**Authors:** Frederick Charles Campbell

**Affiliations:** Patrick Johnston Centre for Cancer Research, Queen’s University Belfast, BT9 7AE, United Kingdom

**Keywords:** hippocampus, dementia, diet

## Abstract

**Graphical Abstract:**
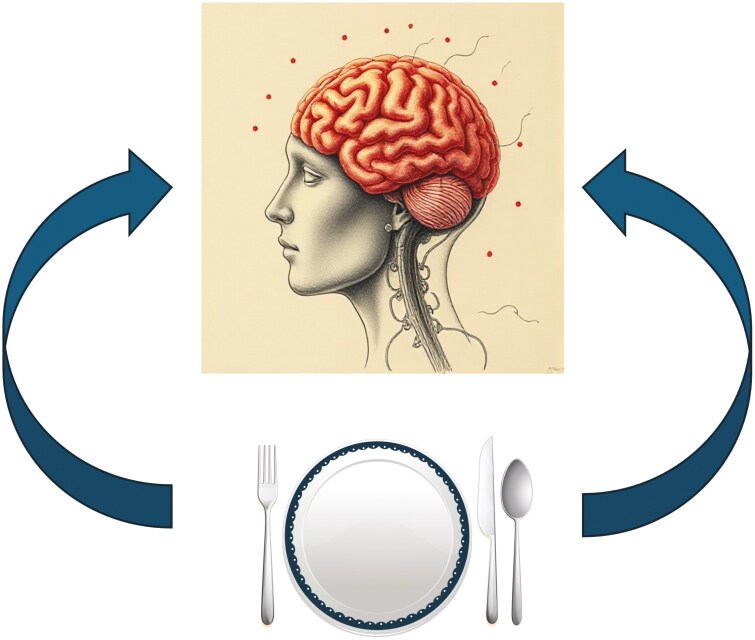

Commentary on **Nutraceuticals: using food to enhance Brain health by modulating postnatal neurogenesis in animal models and patient populations by Ong et al.**

In this issue of the journal, Ong et al consider nutraceutical regulation of adult hippocampal neurogenesis (AHN), as a potential strategy for cognitive decline. The authors seek to enhance understanding of relevant nutraceutical biology and mechanisms implicated in brain health. In this context, the article reviews adult hippocampal stem cell proliferation, cell fate commitment, differentiation, maturation, molecular regulation as well as the role of these processes in cognition. Nutraceuticals are defined as food products with medical benefits^1^ and are categorized in the review as natural food components, functional dietary products, or dietary supplements. The manuscript considers fundamental metabolic effects of caloric restriction or excess, as well as the impact of specific nutraceuticals on cognition, neurogenesis, and associated molecular regulatory networks. The authors consider future nutraceutical therapies for neurodegenerative disease (NDD) against the complexity of bioactive food components, current concepts of molecular regulation, and the potential for toxicity.

Development of novel nutraceutical strategies for cognitive disorders requires a broad understanding of thought, perception, and memory processes. In health, knowledge, attitude, and preferences can be shaped by imprinting everyday life events into memories.^2^ These can then be recalled at a later date, for interpretation of related experiences.^3^ For example, a bleak memory of foot blisters after a poorly prepared country hike can prompt a choice of strong boots for the next attempt! Central to the accommodation and recall of memory is the brain structure called the hippocampus, located within the temporal lobe.^4^ The hippocampus is a vital component of the limbic system that integrates spatial sensory input, pattern recognition, learning, and memory.^5^ Throughout life, these unique functions are sustained by the remarkable process of AHN.^6^ This phenomenon of adult neural stem cell self-renewal and subsequent generation of specialized cell types underlies cognitive health. Within the adult hippocampus, the subgranular zone of the dentate gyrus is a neural stem cell niche. In this location, neural stem cell division, cell fate commitment, and differentiation are modulated by niche-resident cellular populations, vascular elements, scaffolds, and intricate signaling networks. These processes generate new specialized adult neurons that are then integrated into existing hippocampal circuitry^6^ to confer extraordinary brain plasticity for memory, cognition, and learning.^7^

These hierarchical arrangements progressively deteriorate in Alzheimer’s disease (AD),^8^ a devastating NDD and a principal cause of dementia.^9^ Initial stages of AD are characterized by hippocampal atrophy that correlates with impaired formation of new memories, disordered decision making, and overall cognitive decline.^10^ Pathological changes of AD include the formation of β-amyloid peptide (Aβ) plaques, neurofibrillary tangles (NFTs) composed of hyperphosphorylated Tau (pTau),^11^ as well as impaired AHN.^8,12^ These aberrant processes may be interrelated, to the extent that forced overexpression of pTau in murine brain suppresses AHN,^13^ and the level of AHN impairment correlates with Aβ accumulation in an AD transgenic model system.^14^ In humans, Aβ and/or Tau pathologies correlate with cognitive decline, but the relationship is far from inevitable. Some individuals can successfully adapt to AD-associated neurotoxic damage and remain cognitively intact. Such cognitive resilience or “reserve” despite substantive Aβ and/or Tau buildup can be sustained by AHN-mediated replacement of damaged neurons.^15^ Furthermore, cognitive reserve in the face of AD-associated neuropathology is characterized by increased hippocampal neural stem cell number and formation of new neurons.^16^

Poor diet weakens our struggle for the mind as well as the body. Modern “Western type” diet can induce clinical risk factors for AD, including high serum low-density lipoprotein cholesterol, obesity, and diabetes.^17-20^ In recent years, the global incidence of AD has dramatically increased^9^ and over 50 million individuals now live with dementia, our greatest global health challenge.^20^ Health costs for dementia exceeded $800 billion in 2015, and its incidence is expected to increase 3-fold by 2050.^20^ Over 40 years of research has identified active disease mediators^19,21^ as well as various interrelated but modifiable risk factors.^20^ Furthermore, accumulating evidence indicates that a sizable fraction of dementia can be prevented.^20^

To paraphrase Hippocrates, food may shape the mind as well as the body.^22^ Adherence to healthy dietary patterns is associated with a lower risk of dementia, as shown by a meta-analysis of observational studies.^23^ These and other promising findings^24^ have prompted the search for cost-effective nutraceutical strategies as an alternative to new drug development. Proof of principle experiments have shown that nutritional agents can modulate the molecular regulatory framework for AHN.^25^ Furthermore, metabolic changes caused by dietary caloric restriction can induce AHN in AD disease models^26^ and also improve hippocampus-associated cognitive tasks in human subjects.^27^

Despite extensive studies in NDD model systems,^12,14,28^ large-scale clinical nutritional intervention trials for AHN are lacking. Disappointingly, randomized trials of dietary and nutritional supplements against cognitive outcomes in dementia have so far reported null effects.^29^ Consequently, diet has not been considered as a modifiable risk factor for dementia by the Lancet Commission for Prevention, Intervention, and Dementia Care.^20^ Notwithstanding these discouraging events, the scale of the global dementia burden and the lack of any pharmacological solution^20^ necessitate innovative approaches to translate robust observational or experimental findings into effective mechanistic therapy.^29^

Concerns about methodological limitations of previous nutritional clinical trials have prompted recommendations for better use of functional cognitive imaging and novel biomarkers in NDD.^29^ In this context, participant selection could be guided by magnetic resonance imaging of hippocampal volumes and/or APOE ε4, (the Alipoprotein E epsilon 4 allele) a major genetic risk factor for AD. This approach could enable more mechanistic interventions in focused risk subgroups.^29^ Furthermore, biofluid and electrophysiological biomarkers as well as positron imaging tomography evaluations of Aβ accumulation may have an important role in treatment outcome assessment.^30^ This emerging framework for future study design^30^ as well as the novel findings in human disease-relevant model systems^8,28^ give grounds for optimism and lay foundations for a comprehensive program of exciting, precision nutraceutical strategies for AHN.

In their review, Ong et al provide an important understanding of potential mechanisms underlying nutraceutical health benefits. Specifically, they present evidence of nutraceutical effects upon neurogenesis and neurogenesis-associated behavior that will inform future work. Collectively, these steps may set the stage for blockade of causal niche pathways and thus alleviate the devastating and ever-increasing NDD health burden.

## Data Availability

No new data were generated or analyzed in support of this commentary.
